# Patient-specific structural effects on hemodynamics in the ischemic lower limb artery

**DOI:** 10.1038/srep39225

**Published:** 2016-12-15

**Authors:** Pengcheng Xu, Xin Liu, Qi Song, Guishan Chen, Defeng Wang, Heye Zhang, Li Yan, Dan liu, Wenhua Huang

**Affiliations:** 1 Southern Medical University, Institutes of Clinical Anatomy, Guangzhou, 510515, China; 2 Shenzhen Institute of Advance Technology, Research Center for Biomedical Information Technology, Chinese academic of science, Shenzhen, 518055, China; 3 Curacloud Corporation, Center of Medical Image Computing, 999 Third Ave, STE 700, Seattle, WA 98104, USA; 4 Sun Yat-sen Memorial Hospital, Department of Endocrinology, Guangzhou, 510120, China; 5 The Chinese University of Hong Kong, Department of Imaging and Interventional Radiology, Hong Kong, 999077, China

## Abstract

Lower limb peripheral artery disease is a prevalent chronic non-communicable disease without obvious symptoms. However, the effect of ischemic lower limb peripheral arteries on hemodynamics remains unclear. In this study, we investigated the variation of the hemodynamics caused by patient-specific structural artery characteristics. Computational fluid dynamic simulations were performed on seven lower limb (including superficial femoral, deep femoral and popliteal) artery models that were reconstructed from magnetic resonance imaging. We found that increased wall shear stress (WSS) was mainly caused by the increasing severity of stenosis, bending, and branching. Our results showed that the increase in the WSS value at a stenosis at the bifurcation was 2.7 Pa. In contrast, the isolated stenosis and branch caused a WSS increase of 0.7 Pa and 0.5 Pa, respectively. The WSS in the narrow popliteal artery was more sensitive to a reduction in radius. Our results also demonstrate that the distribution of the velocity and pressure gradient are highly structurally related. At last, Ultrasound Doppler velocimeter measured result was presented as a validation. In conclusion, the distribution of hemodynamics may serve as a supplement for clinical decision-making to prevent the occurrence of a morbid or mortal ischemic event.

Lower limb peripheral artery diseases (LLPADs) are prevalent chronic non-communicable diseases that affect approximately 202 million people worldwide^1^. LLPADs have been recognized as the result of insufficient blood supply to the leg, which caused pain and dysfunction. Currently, doctors mostly employ patient questionnaires to distinguish the pain caused by LLPADs from that of other etiologies. Furthermore, researchers have developed a decision-making program for peripheral artery diseases to prevent the occurrence of a morbid or mortal ischemic event^2^. However, many people with LLPADs report no symptoms, even some patients with moderately severe disease. Unfortunately, LLPADs are associated with a very high risk of myocardial infarction, stroke, and death^3^. Early detection of the LLPADs is important, because interventions to promote smoking cessation, lower blood pressure, or offer statin therapy can reduce mortality^4^. Clinically, gold-standard noninvasive identification of LLPADs is ankle-brachial pressure index (ABPI) which requires time, experience and training^5^.

Currently, it is widely accepted that radiological techniques are efficient tools for investigating the anatomy of lower limb arteries. Computed tomography (CT) has high contrast and spatial resolution^6^. Vukram proposed a preprocedural assessment that uses multidetector computed tomography to evaluate the prevalence of significant peripheral artery disease^7^. However, CT scanning can often misrepresent the extent of luminal stenosis based on the silhouette of the curvature. Magnetic resonance imaging (MRI) technology provides another noninvasive method for assessing the morphological features of atherosclerotic arteries^8^. In addition, ultrasound imaging is widely employed for surgical guidance for femoral artery disease operations^9^,^10^. Ultrasonic Doppler velocimeter (UDV) and laser Doppler velocimeter (LDV) measurements are commonly used in clinical diagnoses of ischemia. Ultrasound Doppler is not only useful in the observation of static contractions, but it has also been validated to estimate femoral artery blood flow during exercise^11^.

However, the measurement of radiological techniques only shows a local view. A more complete view is preferred in both research and clinical diagnostic applications. Computational fluid dynamics (CFD) is one effective tool for studying the blood flow patterns inside blood vessels^12^. The detailed characteristics of arterial flow patterns can reflect the vascular features. The relationship between flow pattern and disease can demonstrate the pathogenesis or severity of the illness. CFD is a finite element approach that has been used to describe the flood due to its high flexibility for irregular geometry^13^. The method can solve clinically relevant blood flow problem and test hypotheses regarding hemodynamics factors in vascular adaption and disease. Morishita developed a non-invasive evaluation of abdominal aortic properties^14^. Using a combination of MRI and CFD techniques, Wood and colleagues described a risk factor for the development of LLPADs^15^. A later study showed hemodynamic analysis of the femoral artery bifurcation^16^. An appropriate boundary condition can clearly simplify the calculating process. Willeme improved the inlet boundary conditions to provide more convinced results by using realistic values^17^. The structured-tree model and Windkessel (WK) model are prevalently used in current CFD simulation because of their accuracy^18^. However, the computational cost of the WK model is significantly lower. Ye reported that the simplification of the WK model often ignores the nonlinear and convective term of the Navier-Stokes equations, resulting in errors in the parameter values^19^. Therefore, the authors considered the effects of the vessel taper and compliance when calculating the vessel resistance. Another group’s investigation provided appropriate outflow 0-D models for patient-specific simulation^20^. Their study demonstrated good numerical convergence and stability. In conclusion, CFD provides us visual distributions of the hemodynamics. While certain types of the hemodynamic stresses are essential for physiological function of the endothelial cells under normal conditions, other types can induce endothelial dysfunction by adversely modulating endothelial signaling and gene expression, thus contributing to the development of vascular pathologies^21^. As a result, an experienced doctor may predict the development of LLPADs through the hemodynamic distribution. Moreover, the guiding role of the hemodynamic distribution in hemodialysis has been widely accepted. Therefore, a reliable CFD simulation might have great potential to assist therapy decision making in the future.

We developed a patient-specific lower limb peripheral artery model to assess the status of the blood vessel comprehensively. The computational fluid dynamics were simulated using patient-specific geometries to obtain the distributions of the velocity, pressure, and wall shear stress. Our study showed hemodynamic distribution of typical geometric features, including stenosis, bending, bifurcation, and their combined effects. Moreover, the UDV measured velocities were obtained as the validation. Combining the prior studies, the assessment of hemodynamics may serve as a supplement for treatment decision-making to avoid the overestimation or underestimation of the anatomical assessment.

## Results

The CFD simulation was performed on 14 femoral arteries of seven patients. UDV-measured results were prioritized for comparison and verification. We simulated three flow cycles so the result would meet the criteria of convergence.

### 3D geometric models

**Figure 1:**
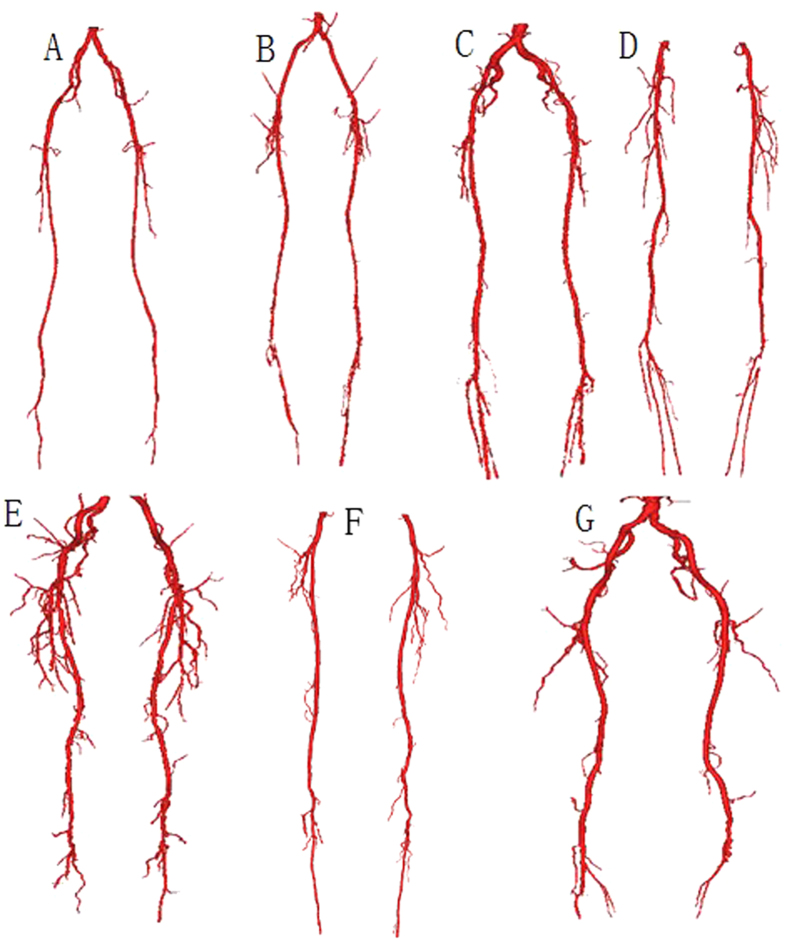
Visual illustration of the patient-specific geometrical models. These models were reconstructed from the lower limb arteries of the seven patients. The reconstructed geometric models differed from each other, according to a difference in MRI data acquisition.

The patient-specific geometries were reconstructed based on MRI images. According to the differences in data acquisition, the reconstructed models contain different parts of the peripheral arteries. A total of seven patients and fourteen vessels were investigated for simulation. An overview of 3D geometric models was shown in Fig. 1. As showed in Fig. 1F, the most proximal portion of the blood vessel only visualizes the common femoral artery. In contrast, the blood vessel showed in Fig. 1G nearly extended to the abdominal aorta. The distal aspect of the vessels varied from Fig. 1A–G. Figure 1E showed great detail in vessel branching, whereas Fig. 1F shows very little branching at the distal end. The total length of one femoral artery of the geometric model is 70 mm (±18.8 mm). The diameters of the inlet and main outlet (largest outlet) are 6.75 mm (±0.53 mm) and 7.5 mm (±3.36 mm), respectively. Stenosis was easily observed in some geometric models (Fig. 1B–E,G) but was not significant in other models. The features of the geometric models are shown in Table 1.

### Mesh generation

**Figure 2:**
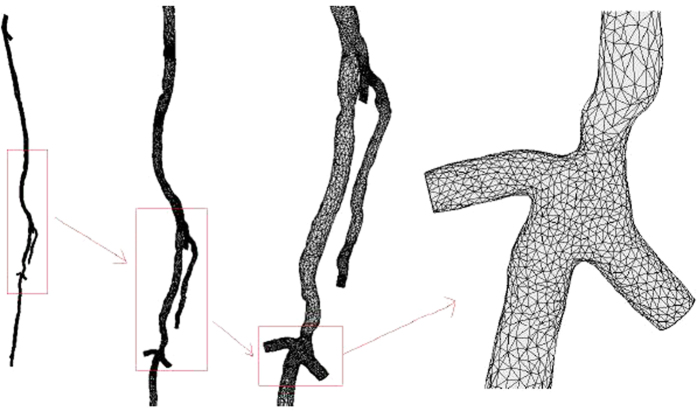
Visual illustration of the mesh-generated result of one lower limb artery from one patient. Panels shown from left to right are increasingly zoomed views of the mesh.

**Figure 3:**
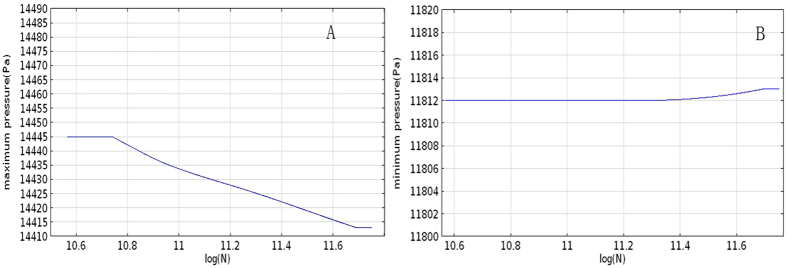
Density test of the mesh for CFD simulation. The maximum pressure of the simulation calculated by varying density is shown in **A**. The minimum pressure of the simulation calculated by varying density is shown in **B**.

The mesh generations based on the 3D geometric models, but they were different. The main purpose of generating a mesh was that it effectively represents the blood structure and is easy to calculate. As a result, the microcirculation and distal blood vessels were ignored in the mesh, as showed in Fig. 2. For the unification of the calculation, all models were divided into two “numerical” domains, each containing DFA, SFA and PA. The total number of elements was 46186, including 24424 triangular elements. The mesh volume was 4.471 × 10^−5^ m^3^. The edge of the triangle became shorter as the radius of the lumen was reduced. A test of the mesh density is shown in Fig. 3. In this mesh test, the lowest mesh density responded to the quality measure threshold at 0.4. As showed in Fig. 3, the maximum pressure was more significantly affected by the mesh density, but the total change was no more than 0.22% when the density was nearly doubled.

### Hemodynamics distribution

Three typical cases were selected to show the detailed hemodynamic distribution. In the first case, the radius of nearly the entire popliteal artery was significantly smaller than that of the deep femoral artery. The second case had nearly isolated structural characteristics. In the third case, the combination of the structural features played an important role in hemodynamics distribution.

**Figure 4:**
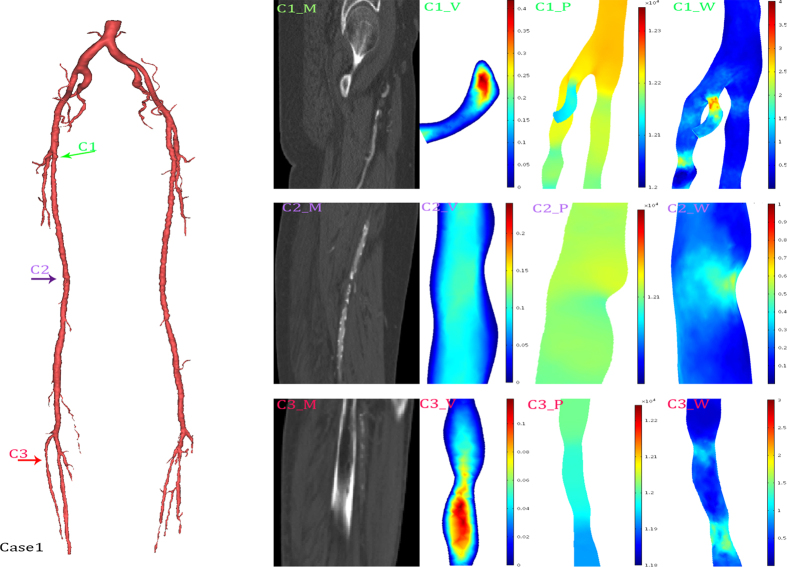
Hemodynamics distributions of the case 1. The reconstructed model in 3D (left), and its typical geometrical features were marked with colored arrows. The first column (C1_M, C2_M, C3_M) were MRI data; the second column (C1_V, C2_V, C3_V) were slice rendered velocity distribution, the unit of the color bar was m/s; the third column (C1_P, C2_P, C3_P) were pressure distribution, the unit of the color bar was Pa; the fourth column (C1_W, C2_W, C3_W) were distribution of the mean diastolic wall shear stress, the unit of the color bar was Pa. Each row described the same geometrical feature marked in the 3D model and MRI data.

**Figure 5:**
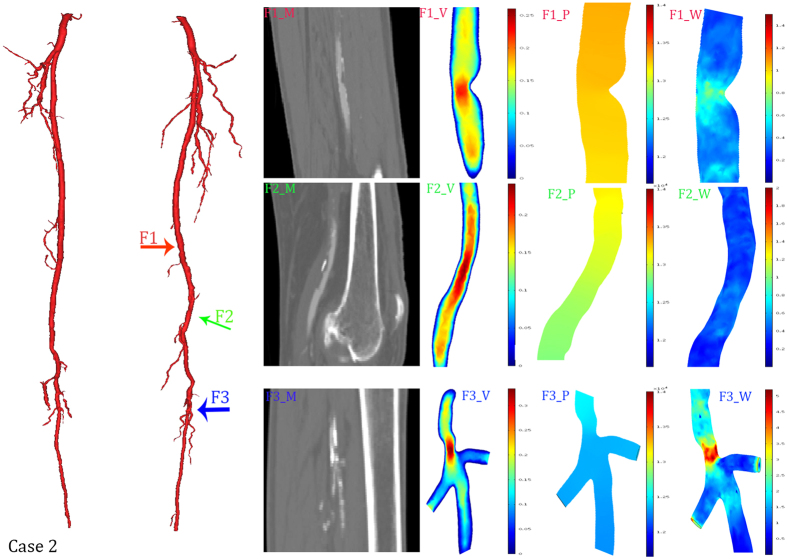
Hemodynamics distributions of the case 2. The reconstructed model in 3D (left), and its typical geometrical features were marked with colored arrows. The first column (F1_M, F2_M, F3_M) were MRI data; the second column (F1_V, F2_V, F3_V) were slice rendered velocity distribution, the unit of the color bar was m/s; the third column (F1_P, F2_P, F3_P) were pressure distribution, the unit of the color bar was 10^4^ Pa; the fourth column (F1_W, F2_W, F3_W) were distribution of the mean diastolic wall shear stress, the unit of the color bar was Pa. Each row described the same geometrical feature marked in the 3D model and MRI data.

**Figure 6:**
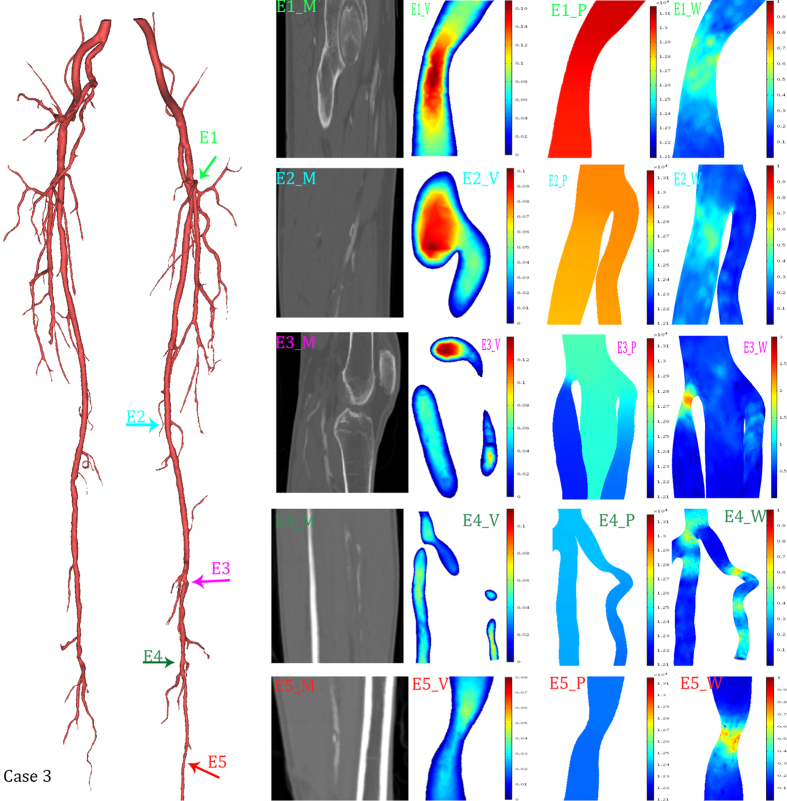
Hemodynamics distributions of the case 3. The reconstructed model in 3D (left), and its typical geometrical features were marked with colored arrows. The first column (E1_M, E2_M, E3_M, E4_M, E5_M) were MRI data; the second column (E1_V, E2_V, E3_V, E4_V, E5_V) were slice rendered velocity distribution, the unit of the color bar was m/s; the third column (E1_P, E2_P, E3_P, E4_P, E5_P) were pressure distribution, the unit of the color bar was Pa; the fourth column (E1_W, E2_W, E3_W, E4_W, E5_W) were distribution of the mean diastolic wall shear stress, the unit of the color bar was Pa. Each row described the same geometrical feature marked in the 3D model and MRI data.

A visualization of the hemodynamic descriptors in representative models was shown in Figs 4, 5 and 6. It can be observed that the geometrical features play important roles in hemodynamics distribution. All these three figures were shown an instantaneous result. As showed in Fig. 5, three typical features were selected to describe the structural effects. The green arrow (C1) directed to a bending side branch. Significant velocity and wall shear stress increase, 0.4 m/s and 3.7 Pa, respectively, were found in the bending side branch. The pressure gradient also increased in the side branch. The purple arrow and red arrow both directed the stenosis while the red arrow directed the stenosis at the narrow popliteal artery. According to our results, the similar degree of the stenosis at the different location led to many different alteration of the hemodynamics. The stenosis (23%) at the normal lower limb artery caused a 0.4 Pa WSS increase and no significant velocity increase. In contrast, the similar stenosis (25%) at the narrow lower limb artery caused a 1.5 Pa WSS increase and 0.1 m/s velocity increase.

Figure 5 described three nearly isolated geometrical features of the lower limb artery. Compared to the case1, the alteration of the pressure gradient can be neglected in case2. With the increasing severity of the stenosis, the stenosis became the dominant factors of hemodynamics distribution. A 28% stenosis (marked by red arrow F1) caused a 0.1 m/s velocity increase and 0.7 Pa WSS increase. The 26° bending (marked by blue arrow) and little narrowed trifurcation (marked by green arrow) made similar difference to the velocity alternation. The little narrowed trifurcation caused the maximum WSS increase, 5.2 Pa, while the bending did not cause significant changes.

Figure 6 was presented to show the combined effects of the geometrical features. The 46° bending near the bifurcation (marked by dark green arrow E4) caused an increment of 0.7 Pa. In contrast, the isolated bending (marked by green arrow E1) and bifurcation (marked by light blue arrow E2) caused increases of 0.3 Pa and 0.5 Pa, respectively. Moreover, along the curve approaching the bifurcation, obvious fluctuation altered the distribution of the WSS. The stenosis at the bifurcation (marked by purple arrow E3) caused an increment of 2.7 Pa. By contrast, the isolated stenosis (marked by red arrow E5) and bifurcation caused increases of 0.7 and 0.5 Pa, respectively.

**Figure 7:**
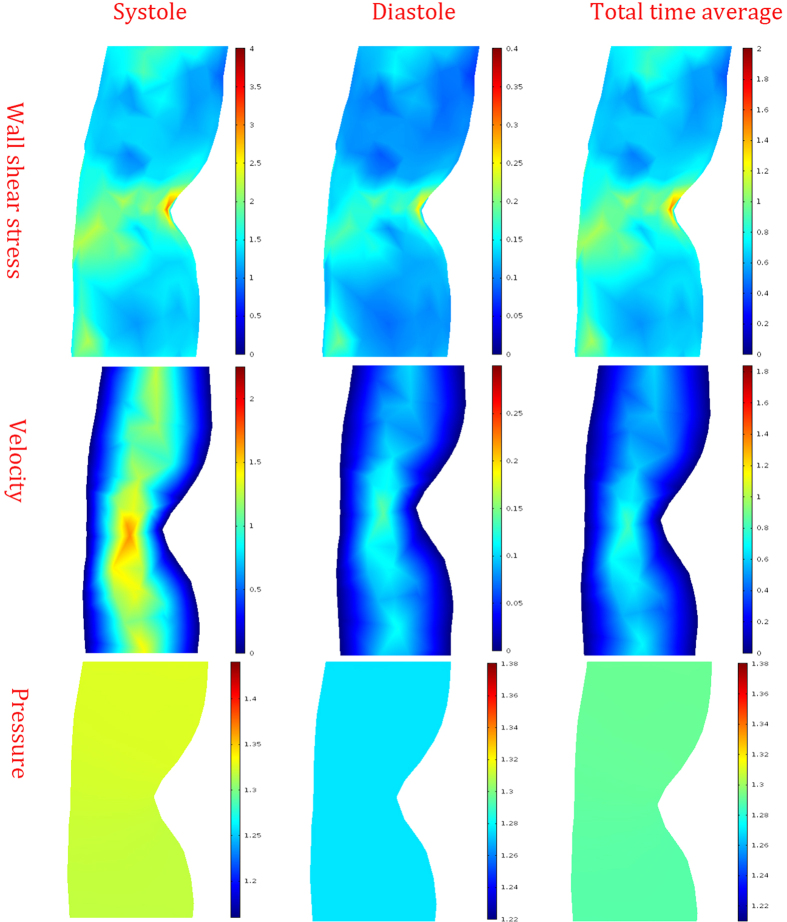
Hemodynamic distribution of different periods. The distributions of the wall shear stress were showed in the top column, the unit was Pa. The distributions of the velocity were showed in the middle column, the unit was m/s. The distributions of the pressure were showed in the bottom column, the unit was 10^4^ *Pa*.

Additionally, the hemodynamic distribution of different period was showed in Fig. 7. According to the result, the peak systolic WSS was 10 times larger than that of diastole. Similar effects were observed in velocity and pressure distribution. Although there was a large difference in the values of the parameters, the impacts of the geometric feature on the hemodynamic distribution were almost the same.

For the given models, the hemodynamics distribution of the patient-specific lower limb artery models can be visually observed. These results may become a reliable assistant of doctors.

### Velocity profile

**Figure 8:**
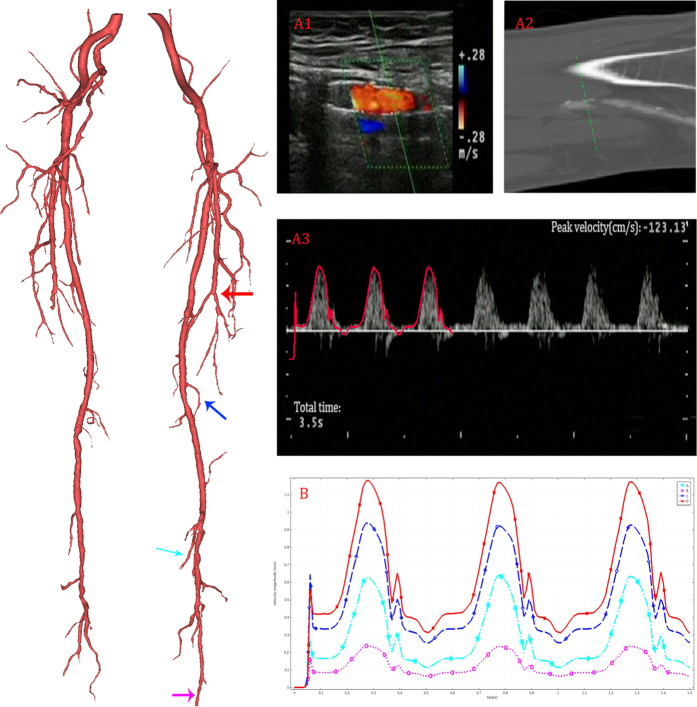
Computational results of velocity profile. The reconstructed model in 3D (left), and UDV measured location was marked in red arrow. The UDV measured result was shown in **A1**. The corresponding location of MRI data was shown in **A2**. In **A3**, the calculated velocity profile (outlined in red) are overlapped on the UDV measurements to validate the accuracy of the simulation. The velocity profiles of four typical outlets (marked by colored arrow in 3D model) calculated by CFD are shown in **B**.

The comparison of the UDV measurement and CFD result was presented in Fig. 8. Figure 8A1 described the same location of the femoral artery marked by red arrow in 3D model. The UDV-measured (A1) data and MRI (A2) data were clinical data acquired by one experienced doctor. In Fig. 8A3, the calculated velocity profile of the femoral artery (in red) is overlaid on the UDV-measured result to verify the accuracy of the simulation. The measured location had been shown in A1 (A2). The velocity profile of several outlets (marked by colored arrow) of a typical patient-specific model is shown in Fig. 8B. The velocity profiles of the outlets differed. The maximum velocity of the outlet at peak systole was approximately 1.20 m/s, whereas the minimum velocity during the same time was only approximately 0.25 m/s. Although some differences existed, the total tendency of the velocity profile at the outlet exhibited high consistency.

In addition, the statistically analyzed results were shown in Table 2. The correlation coefficient was used to describe the relationship between UDV and CFD results. As the correlation coefficient approaches 1, the described relation becomes stronger. As showed in Table 2, the correlation coefficient of all patient-specific model results and UDV results was greater than 0.9, with a P-value much greater than 0.05. The last term of Table 2 describes the correlation of the total data using a 2-tailed *t*-test with a significance level of 0.01.

## Discussion

We have performed hemodynamic simulation in patient-specific three-dimensional geometric models of the stenotic femoral artery. As showed in Fig. 1, although even the models seemed to show a significant difference, the framework of the models stayed the same. The DFA, SFA, PA branches can be found clearly in all models. Distinct from the arterial tree models, our geometric models showed more detailed structures, which enabled us to study the impact of geometric characteristics on hemodynamics^22^,^23^.

The pressure gradient is the most traditional index used to diagnose femoral artery disease. A hyperemic gradient of >25 mmHg compared with control is considered an indication of artery disease^24^. Our study also showed an obvious increase of the pressure gradient at the stenosis. Furthermore, the difference in systolic blood pressure was reported related to vascular disease and mortality^25^.

WSS has been widely accepted as an important factor in the development of arterial atherosclerosis^16^,^26^,^27^,^28^,^29^,^30^,^31^,^32^. Clinically, the WSS attracted more and more attention in therapy decision-making program for LLPADs. This topic was raised by many international academic conferences. Moreover, Malek and colleagues showed that arterial level shear stress (>15 dyne/*cm*^2^) induces endothelial quiescence and an atheroprotective gene expression profile, whereas low shear stress(>4 dyne/*cm*^2^), which is prevalent at atherosclerosis-prone sites, stimulates an atherogenic phenotype^33^. Glagove illustrated the strong relation between shear stress and atherosclerosis, which is the main cause of LLPADs^34^. In general, high WSS will easily lead to the rapture of the calcified plaque, and the low WSS region is ideal depositing position. Additionally, the normal type can easily induce the endothelial dysfunction. The ABPI or questionnaire was hardly to obtain the location of the dysfunctional vascular vessel. While our study can easily observe the occlusive arteries and abnormal WSS place. The biggest advantage of our study is forecasting. Combined with clinical research^4^,^5^,^21^ and hemodynamic distribution, doctors can predict the development LLPADs. An early detection can help patients managing risk factors through exercise, a healthy diet, smoking cessation, and medical treatment.

As showed in Figs 4, 5 and 6, WSS significantly increased in stenosis and bifurcations, and moderately increased in the bending of the blood vessel. The WSS alteration caused stenosis and bifurcation nearly as twice as much as it caused bending. Therefore, vascular bending rarely raised attention until Wood noted that the curvature is a possible risk factor^15^. Compared to Wood’s work, our study showed a more specific model. In other words, our CFD simulation provided more compelling results. A recent study with an overview of the full-body arterial network showed high WSS in vessels with a narrow lumen^35^. The instantaneous WSS contours in Kim’s work showed that WSS increases at the bifurcation. We also validated that bifurcations play an important role in WSS distribution in patient-specific models. Moreover, many other studies have confirmed a similar impact of the bifurcation^36^,^37^. Moreover, we proved that geometric features (including stenosis, bifurcation and bending) could synergistically affect WSS. The changing of the WSS distribution can easily lead to the rapture and deposition of the calcified plaque. Additionally, the calcified plaque is the main cause of the atherosclerosis. As a consequence, bifurcation is the risk region that tends to occur significant WSS alteration^21^. This may explain why atherosclerosis most commonly occurs in the bifurcation^38^.

However, the precise relationship of geometric characteristics and WSS alteration remains elusive. We have not yet found a quantitative relation between them, although the analysis of WSS of the personalized model is good guidance for the diagnosis of LLPADs.

Earlier experimental studies have revealed that the pathology of atherosclerosis relates to the flow disturbance. Velocity distribution can be a positive way to describe flow disturbance, which has a strong correlation with the development of atherosclerosis^39^. As our results show, the change in velocity mainly occurs in the stenosis and bifurcation. Wood and colleagues showed a strong correlation between tortuosity and flow disturbance in femoral arteries^15^. Investigating cycled velocity variation can easily determine the flow disturbance. By combining the investigation of the flow disturbance and previous clinical studies, physicians may be able to reveal LLPADs in a timely manner and thereby prevent deterioration. Moreover, the hemodynamic distribution may also work for vascular surgery^40^.

The statistical analysis and comparison result proved that there was no significant difference between the CFD results and UDV measurements. In other words, stable computational hemodynamic indices may become reliable references in subsequent clinical trials.

## Limitation and Conclusion

As mentioned before, the WK model is appropriately applied in the microcirculation or distal blood vessels, in which the radius of the lumen is very small. When coupling the lumped parameter model to the three-dimensional geometry model, we truncate the collateral blood vessel without considering whether the radius is small enough to use these outflow boundary conditions. Subsequent studies will focus on the radius when WK models are appropriate for coupling to the large artery.

Another limitation was the statistical analysis applied in this study. There were not enough samples (only six sample velocities per patient) due to insufficient clinical data. While statistical analysis is ideally based on a large number of experimental replicates, usually N > 50, fewer replicates may lead to inaccuracies. Therefore, it is necessary to obtain more data for further validation in future studies.

In conclusion, our study developed one patient-specific lower limb artery models to evaluate blood flow properties. The UDV-measured velocity was obtained prior as the comparison and validation method for CFD simulation. The effect of geometric factors on the distribution of hemodynamics was presented in this study. The velocity decreased as the flow left the inlet and increased when the radius of the lumen reduced. Stenosis, bifurcation and vessel curvature played important roles in the increases in pressure gradient and WSS. Moreover, the combination of the risk factors can lead to a more hostile hemodynamic environment than the risk factors can individually. The statistical analysis and velocity profiles demonstrate the reliability of our study. In conclusion, stable CFD simulation has the potential to become a reliable supplement for therapy decision-making programs.

## Method

### DATA acquisition and mesh generation

This study was conducted in accordance with the principles of the Declaration of Helsinki and met the requirement of medical ethics. The Ethical Review Committee of the Sun Yat-sen Memorial Hospital (Guangzhou, Guangdong, China) approved this research. Because our study was purely observational and retrospective in nature and used anonymized data, patient approval and informed consent were waived. Patient-specific data sets were provided by the Sun Yat-sen Memorial Hospital (Guangzhou, Guangdong, China) and included both MRI and ultrasound data. The data set acquisition focused on lesions, so the investigative data were varied across patient. However, all MRI data included the main branch of the lower limb arteries that span from superficial arteries to popliteal arteries. Therefore, the geometric models all included the following lower limb arteries: Deep Femoral Artery (DFA), Superficial Femoral Artery (SFA), and Popliteal Artery (PA). Ultrasound data were obtained at the main branch and region of stenosis. The entire lower limb 3D models were reconstructed by the commercial imaging processing software Mimics (Materialise, Leuven, Belgium (trial version)) using lower limb MRI images. The mesh generations were accomplished corresponding to the reconstructed 3D geometric models. According to the work of Szczerba and Shewchuk, we manually adjusted the auto-generating mesh with a quality measure threshold of 0.4^41^,^42^. Mesh density is directly related to the quality measure threshold; therefore, the higher the quality is, the greater mesh density will be. A test of the mesh density was conducted to prove the appropriate quality measure threshold. Each individual varied in meshing elements. The number of the elements varied from more than 40,000 to 120,000 because of the patient-specific models. The total mesh volume was approximately 5 × 10^−5^ m^3^.

### Computational Fluid Dynamics (CFD)

Computational fluid dynamics (CFD) is a branch of fluid mechanics that uses numerical analysis to solve physics problems involving fluid flow. CFD is an efficient method for analyzing the hemodynamics of the vasculature. A valid result would describe the distribution of the flow velocity, wall shear stress and pressure in the femoral artery. We separated the spatial domain into a “numerical” domain and an “analytical” domain, referring to the coupled multi-domain method^43^. The domains are separated by the interface Γ. The “numerical” domain corresponds to the 3D geometry that is reconstructed from clinical MRI data for which the Navier-Stokes equations were employed. We assumed that blood can be described as an incompressible Newtonian fluid with a blood viscosity of 0.004 Pa·s and a density of 1056 kg/*m*^3^. We assumed that the wall was rigid without slipping. Therefore, the governing equation can be expressed as follows:


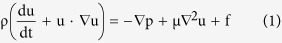



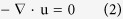


where the density of blood, ρ, is assumed to be constant at 1056 kg/*m*^3^; **u** is the flow velocity field; p is the pressure; F is the body force, which is taken to be zero; and μ is the flow viscosity. The “analytical” domain corresponds to the distal vascular networks and microcirculation that are not included in the 3D geometric model below and whose physics are described in lumped-parameter models.

#### Outflow Boundary conditions

When the radius of the distal vascular network is sufficiently small, the ratio of the flow velocity to the pulse wave speed and the variation in the pulse wave speed both become very small^44^,^45^. Hence, the relation between blood pressure and flow rate should be linear. The Windkessel model, also called the elastic reservoir model, is one of the earliest mathematical theories to describe gross pulsatile behavior^46^. This model was inspired by the elastic reservoir system equation and gave a convenient way to consider the electrical analog of the vascular system to draw the analogies described in Table 3. The studies used the current (*i*_*in*_, *i*_*out*_) to describe the flow rate (*f*_*in*_, *f*_*out*_), the voltage (*v*_1_, *v*_2_) to describe the pressure (*p*_1_, *p*_2_), the capacitance (*C*_1_, *C*_2_) to describe the compliance (*K*_1_, *K*_2_), the inductance (L) to describe the mass equivalent (M) and the resistance (R) to describe the peripheral resistance (r).

**Figure 9:**
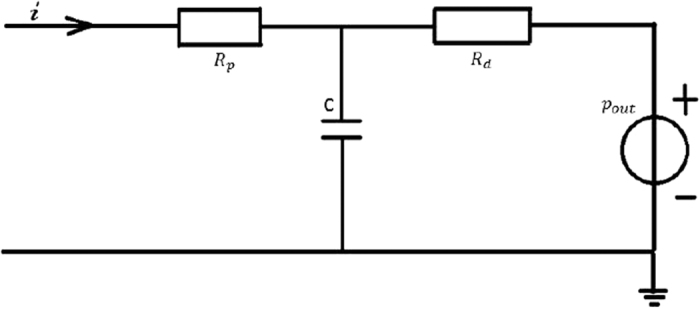
The schematic illustration of the Windkessel model. The parameters were defined as follows: *R*_*p*_ represents the proximal resistance, *R*_*d*_ represents the distal resistance, C represents the compliance of the artery, *p*_*out*_ represents the pressure in the realistic terminal, and *i* represents the flow rate at the boundary outflow.

Our study implemented an improved three-element WK model, as shown in Fig. 9. We assumed that blood can be described as an incompressible Newtonian fluid with a blood viscosity of 0.004 Pa·s and a density of 1056 kg/*m*^3^. We assumed that the wall was rigid without slipping. As shown in Fig. 9, *R*_*d*_ represents the distal resistance, R_*p*_ represents the proximal resistance, (*R*_*p*_ + *R*_*d*_) is equal to total peripheral resistance *R*_*T*_, and the ratio of proximal to total resistance is assumed to be 0.056^35^. C represents the compliance of the artery. *ρ*_*out*_ is assumed to be constant at 2.6 KPa. In addition, *R*_*T*_ is defined as follows:


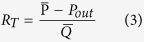


where 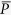 is the mean pressure in the microcirculation, *P*_*out*_ is the pressure at the physical outlet boundary, and 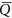 is the mean flow rate in the lumen.

With the help of Kirchhoff’s law, the equation of microcirculation was described as follows:


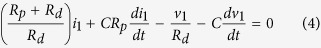


where *i*_1_ is the outflow rate, *v*_1_ is the unknown pressure, *R*_*d*_ is the distal resistance, *R*_*p*_ is the proximal resistance, and C is the compliance of the artery.

#### Boundary conditions at the inlet

The waveform of velocity at the inlet, as derived from MRI images, was implemented as the inlet boundary condition for the 3D geometric model. The inflow is described as


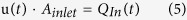


where u(t) is the waveform of the velocity at the inlet, *A*_*inlet*_ represents the cross section area, and *Q*_*In*_(*t*) represents the fluid inflow.

### Wall shear stress

Shear stress is the component of stress coplanar with a material cross section and arises from the force vector component parallel to the cross section^26^. Shear stress is not only a critical determinant of vessel caliber but is also implicated in vascular remodeling and pathobiology^33^. The disturbances caused by abnormal vascular morphology result in decreased wall shear stress are associated with atherosclerosis^47^. In this study, the wall shear stress was calculated according to the following formula:


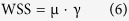


where μ is the dynamic viscosity and γ is the shear rate.

### Statistical analysis

Statistical analysis was applied to prove the effectiveness of the patient-specific CFD simulations. We extracted the blood velocity of several typical locations in both the simulation and clinical data for each patient. Paired-sample *t*-testing was used to examine each result of the seven patient-specific models. The Pearson correlation coefficient was calculated to examine the total relation between the UDV-measured and CFD-calculated results.

## Additional Information

**How to cite this article**: Xu, P. *et al*. Patient-specific structural effects on hemodynamics in the ischemic lower limb artery. *Sci. Rep.* **6**, 39225; doi: 10.1038/srep39225 (2016).

**Publisher's note:** Springer Nature remains neutral with regard to jurisdictional claims in published maps and institutional affiliations.

## Figures and Tables

**Table 1:** Features of the patient-specific models.

Feature	Reduction of the radius by 20–40%	Reduction of the radius by >40%	Max. bending (degree)	Number of branches	Age (year)	Sex	Max. stenosis
Model							
A	2	0	45.16	14	69	m	20%
B	4	2	20.64	12	72	m	95%
C	2	1	28.41	15	80	f	40%
D	1	1	43.48	18	74	m	100%
E	5	1	55.03	36	58	m	85%
F	2	1	54.98	20	79	m	50%
G	2	1	31.85	22	72	m	50%

Four geometrical features are presented: moderate stenosis (reduction of the radius by 20–40%), serious stenosis (reduction of the radius by >40%), maximum bending and the number of branches. Three clinical indicators are presented: age(year), sex (m = male, f = female), degree of stenosis (100% stenosis means completely blocked).

**Table 2:** Paired sample *t* **-**test of the UDV-measured velocity and CFD-calculated velocity results.

Patient	A	B	C	D	E	F	G	Total
Correlation	0.997	0.936	0.994	1.000	0.999	1.000	0.979	0.998
P-value	0.583	0.681	0.764	0.613	0.427	0.910	1.000	0.483

The data of seven patients were examined independently first and then together. The individual examinations are shown in columns A–F, and the total correlations are shown in the last column.

**Table 3:** Detailed electrical analog of the blood flow circulation system.

Physical model	Electrical analog
Flow rate (*f*_*in*_, *f*_*out*_)	Current (*i*_*in*_, *i*_*out*_)
Pressure (*p*_1_, *p*_2_)	Voltage (*v*_1_, *v*_2_)
Compliance (*K*_1_, *K*_2_)	Capacitance (*C*_1_, *C*_2_)
Mass equivalent (M)	Inductance (L)
Peripheral resistance (r)	Resistance (R)

The analogies include the main parameters of the physical model, i.e., the flow rate, pressure, compliance, mass equivalent and peripheral resistance.
